# The relationship between intradialytic hypotension and vascular calcification in hemodialysis patients

**DOI:** 10.1371/journal.pone.0185846

**Published:** 2017-10-19

**Authors:** AJin Cho, Young-Ki Lee, Jieun Oh, Jong-Woo Yoon, Dong Ho Shin, Hee Jung Jeon, Myung-Jin Choi, Jung-Woo Noh

**Affiliations:** 1 Department of Internal Medicine, Kangnam Sacred Heart Hospital, Hallym University, Hallym University College of Medicine, Seoul, Korea; 2 Department of Internal Medicine, Kangdong Sacred Heart Hospital, Hallym University College of Medicine, Seoul, Korea; 3 Department of Internal Medicine, Chuncheon Sacred Heart Hospital, Hallym University College of Medicine, Chuncheon, Gangwon-do, Korea; Hospital Universitario de la Princesa, SPAIN

## Abstract

**Background:**

Vascular calcification is associated with structural and functional abnormality of the heart and blood vessels. We investigated the relationship between intradialytic hypotension (IDH) and vascular calcification in hemodialysis (HD) patients, and their impacts on cardiovascular events (CVEs).

**Method:**

We enrolled 191 maintenance HD patients who underwent plain abdomen radiography for abdominal aortic calcification score (AACS). A nadir systolic blood pressure (BP) < 90 mm Hg or the requirement of bolus fluid administration was required to quantify the hypotension diagnosis. IDH was defined as > 2 hypotension episodes during 10 HD treatments.

**Results:**

Among the 191 patients, IDH occurred in 32. AACS was higher in the IDH group compared with the no-IDH group (8.4 ± 6.0 vs. 4.9 ± 5.2, respectively; P = 0.001). High AACS was an independent risk factor after adjustment for age, diabetes mellitus, ultrafiltration, diastolic BP, and calcium level (odds ratio (OR) = 1.09, 95% CI = 1.01–1.18; P = 0.03). Patients with both IDH and AACS ≧ 4 had the highest cumulative CVE rate (27.9%, P = 0.008) compared with 11.2%, 12.5%, and 6% for those with AACS ≧ 4 only, with IDH only, and neither, respectively. In multivariate analysis, the presence of both IDH and AACS ≧ 4 was a significant predictor of CVE (hazard ratio (HR) = 2.84, 95% CI = 1.04–7.74, P = 0.04).

**Conclusion:**

IDH is associated with abdominal aortic calcification and is an independent risk factor for IDH. Both IDH and high AACS were significant predictors of CVE.

## Introduction

Intradialytic hypotension (IDH) is a common complication during hemodialysis (HD). The National Kidney Foundation Kidney Disease Outcomes Quality Initiative (KDOQI) defines IDH as “a decrease in systolic BP by ≥ 20 mm Hg or a decrease in MAP [mean arterial pressure] by 10 mm Hg associated with symptoms that include: abdominal discomfort; yawning; sighing; nausea; vomiting; muscle cramps; restlessness; dizziness or fainting; and anxiety” [1]. On the basis of the KDOQI definition, IDH occurs in approximately 20%–30% of HD sessions [2].

IDH continues to be a leading problem, especially in the cardiovascularly compromised patients. This predominance can be explained by the fact that structural and functional abnormalities of the heart and blood vessels increase patient sensitivity to changes in fluid status [3]. Vascular calcification is common in end-stage renal disease and is associated with cardiac changes [4]. It induces stiffening of the vessel wall and reduces vascular compliance, which has been found to predict cardiovascular mortality. Previous studies have reported that diastolic left ventricular (LV) dysfunction may be induced by increased vascular calcification in chronic HD patients [5].

IDH not only causes discomfort, but also increases patient mortality and cardiovascular events (CVEs) [6–8]. Vascular calcification induces a reduction in vascular compliance and diastolic LV dysfunction. Therefore, IDH may be associated with vascular calcification in HD patients.

This study aimed to determine the relationship between IDH and abdominal aortic calcification evaluated by plain abdomen radiography in HD patients. In addition, the clinical impact on CVEs was evaluated.

## Methods

### Study population

This study was conducted at Kangdong Sacred Heart Hospital, Kangnam Sacred Heart Hospital, and Chuncheon Sacred Heart Hospital. One hundred and ninety-one maintenance HD patients (49 men, 142 women) were eligible between April 2014 and April 2016. HD was performed thrice weekly (for 4 hours/day). Subjects had each received > 3 months of outpatient HD at one of the participating centers and underwent plain abdominal radiography for abdominal aortic vascular calcification. This prospective, observational study was approved by the hospitals’ Institutional Review Boards. Written informed consent was obtained from all patients before enrollment.

### Data collection and definition

Baseline characteristics, including demographics, medical history, laboratory data, and HD data, were collected at the time of IDH assessment. Echocardiography, plain abdominal radiography, and PWV measurement were carried out across one week at the time of IDH assessment. Blood was drawn before starting each dialysis session. Body mass index (BMI) was calculated as the ratio of weight in kilograms divided by the square of height in meters. Mean pre-HD systolic and diastolic blood pressure and ultrafiltration were calculated as the average of 10 values at the time of IDH assessment. CVE was defined as a history of coronary artery disease, arrhythmia, cerebrovascular disease, or peripheral vascular disease; coronary artery disease was defined as a history of angioplasty, coronary artery bypass grafting, myocardial infarction, or angina; cerebrovascular disease was defined as a previous history of transient ischemic attack, stroke, or carotid endarterectomy; and peripheral vascular disease was defined as a history of claudication, ischemic limb loss and/or ulceration, or peripheral revascularization procedure. A nadir SBP < 90 mm Hg or the requirement for bolus fluid administration was required to quantify the hypotension diagnosis. IDH was defined as > 2 episodes hypotension during 10 HD treatments.

### Outcome measures

The primary study outcome was the association between IDH and vascular calcification. The secondary outcome was new-onset CVE. Vascular calcification was scored by abdominal aortic calcification score (AACS) as described by Kauppila et al [9]. A lateral radiograph of the abdomen was performed in a standing position and the aorta was identified as the tubular structure coursing in front of the anterior surface of the spine. We used a semi-quantitative scoring system; only the abdominal aorta segments in front of the first to fourth lumbar vertebrae were considered. Points were assigned on a 0–3 scale to areas of calcification identified along the anterior or posterior surface of the aorta (0 = absent; 1 = small; 2 = moderate; 3 = large) according to the length of each calcified plaque with respect to the craniocaudal length of the closet vertebra. Hence, the score could vary from a minimum of 0 to a maximum of 24 points. All radiographs were read by two investigators and a consensus was reached on the interpretation of all films.

Brachial-ankle PWV (baPWV) was measured using a Vascular Profiler 1000 (VP-1000; Colin Co. Ltd., Komaki, Japan). The baPWV was automatically calculated from the distance between two arterial recording sites divided by transit time. Comprehensive echocardiographic measurements were performed using an ultrasound machine (Vivid 7; GE Vingmed ultrasound AS, Horten, Norway) with a 2.5 MHz probe, based on the imaging protocol by the American Society of Echocardiography guideline.

### Statistical analysis

The data are expressed as means ± standard deviations. Comparisons of continuous variables were performed using t-tests. Categorical variables were compared using either chi-square or Fisher’s exact test, as appropriate. For the primary analysis, we performed multivariate logistic regression to test for the independent contribution of vascular calcification (VC) to IDH. The cumulative incidence of CVEs was calculated using the Kaplan–Meier method and compared using the log-rank test. Cox proportional hazard model was used to identify the independent variables related to CVEs. Baseline associations were estimated by demographic factors and comorbid conditions. The factors might confound associations with outcomes of t testing and chi-squared testing. Associated confounder and clinically important factors were included in univariate analyses. All variables with p-values less than 0.05 in the univariate analyses were included in the multivariate models. All calculations were performed using SPSS 18.0 (SPSS Inc. Armonk, NY). P < 0.05 was considered statistically significant.

## Results

The 191 subjects’ mean age was 60 ± 12 years and 142 (74.3%) were women. One hundred three (53.9%) had diabetes mellitus and 92 (48.2%) had a previous history of CVE. IDH occurred in 32 of the 191 subjects. Their mean ultrafiltration was 2.6 ± 1.0 L and mean AACS score was 5.5 ± 5.5. Demographic and biochemical parameters of all subjects, with and without IDH, are in Table 1.

**Table 1:** Baseline demographics and biochemical variables in study population according to IDH.

Variables	IDH	No IDH	p- value
	N = 32	N = 159	
Demographic data			
Age (years)	62.1 ± 8.0	59.3 ± 12.2	0.2
Male, n (%)	8 (25)	41 (25.8)	1.0
Duration of hemodialysis (months)	65.1 ± 64.9	54.4 ± 54.4	0.3
Diabetes, n (%)	24 (75)	79 (50.6)	0.009
Smoking, n (%)	2 (6.3)	23 (14.5)	0.2
Previous cardiovascular event			
Coronary artery disease, n (%)	14 (43.8)	59 (37.1)	0.5
Peripheral artery disease, n (%)	2 (6.5)	9 (5.7)	0.9
Cerebrovascular accident, n (%)	7 (21.9)	38 (24.2)	0.7
Mean ultrafiltration	2.9 ± 0.8	2.6 ± 1.0	0.03
Mean pre-HD systolic BP	145.4 ± 28.9	152.7 ± 24.5	0.1
Mean pre-HD diastolic BP	72.7 ± 13.8	78.9 ± 11.8	0.02
Antihypertensive medication			
Beta blocker	11 (34.4)	75 (47.2)	0.2
Calcium channel blocker	10 (31.3)	83 (52.2)	0.03
Renin-angiotensin system blocker	18 (56.3)	108 (67.9)	0.2
Laboratory data			
Hemoglobin (g/dL)	10.3 ± 1.0	10.2 ± 1.3	0.6
Albumin (g/dL)	3.7 ± 0.4	3.7 ± 0.4	0.5
Calcium (mg/dL)	8.9 ± 1.0	8.5 ± 0.8	0.03
Phosphate (mg/dL)	4.6 ± 1.3	4.4 ± 1.4	0.3
Cholesterol (mg/dL)	137.3 ± 34.6	136.3 ± 39.3	0.9
Triglyceride (mg/dL)	132 ± 93.2	113.3 ± 69.9	0.2
High-density lipoprotein (mg/dL)	42.6 ± 11.9	42.6 ± 13.2	1.0
Low-density lipoprotein (mg/dL)	75.9 ± 18.0	74.3 ± 25.5	0.7
CRP (mg/L)	4.6 ± 6.5	2.9 ± 7.6	0.3
Parathyroid hormone (pg/dL)	265.3 ± 211.4	331.5 ± 310.2	0.3
Vitamine D use	10 (31.3)	83 (52.2)	0.03
Calcium containing phosphate binder	21 (65.6)	112 (70.4)	0.4
Access type			
Arteriovenous fistula	26 (81.3)	133 (83.6)	0.79
Arteriovenous graft	6 (18.8)	22 (13.8)	
Catheter	0 (0)	4 (2.5)	

Note: values are expressed as median ± SD or median (interquartile range) or number (percentage).

Abbreviations: CRP, C-reactive protein; HD, hemodialysis; BP, blood pressure

IDH occurred in 6.7% of dialysis sessions. No hypotension episode occurred in 129 out of 191 (67.5%) patients while one episode in 30 (15.7%), two episodes in 15 (7.9%), three episodes in 9 (4.7%), four episodes in 3 (16%), five and six episodes in two (1.0%) respectively and seven episodes in one (0.5%) during 10 HD treatments.

Number of hypotensive episodes was linearly associated with vascular calcification (st1306andardized coefficient 0.224, P = 0.002). AACS was higher in the IDH group than the no-IDH group (8.4 ± 6.0 vs. 4.9 ± 5.2, P = 0.001). The median AACS value for all subjects was 4.0. Incidence of AACS ≧ 4 was significantly higher in patients with IDH than in those without IDH (75% vs. 45.9%, respectively; P = 0.003).

We performed logistic regression analysis to identify independent risk factors associated with IDH (Table 2). In univariate analysis, diabetes mellitus (odds ratio (OR) = 3.04, 95% confidence interval, CI = 1.29–7.17; P = 0.01), high ultrafiltration (OR = 1.65, 95% CI = 1.05–2.60; P = 0.03), low diastolic BP (OR = 0.96, 95% CI = 0.93–0.99; P = 0.009), high calcium value (OR = 1.57, 95% CI = 1.02–2.43; P = 0.04), calcium channel blocker (OR = 0.41, 95% CI = 0.18–0.93; P = 0.03), vitamin D use (OR = 0.41, 95% CI = 0.19–0.93; P = 0.03) and high AACS (OR = 1.11, 95% CI = 1.04–1.19; P = 0.001) were significantly associated with IDH. High AACS was an independent risk factor after adjustment for age, diabetes mellitus, ultrafiltration, diastolic BP, and calcium level (OR = 1.09, 95% CI = 1.01–1.18; P = 0.03).

**Table 2:** Logistic models for associating factors of IDH.

Variables	Univariate		Multivariate	
	Odds Ratio (95% C.I.)	p-value	Odds ratio (95% C.I.)	p-value
Age (per year)	1.0(1.0–1.06)	0.2	1.02 (1.0–1.07)	0.5
DM (vs. No)	3.04 (1.29–7.17)	0.01	1.84 (0.7–4.91)	0.2
Ultrafiltration (per 1L)	1.65 (1.05–2.60)	0.03	2.06 (1.16–3.66)	0.01
Diastolic BP (per 10 mmHg)	0.96 (0.93–0.99)	0.009	0.96 (0.92–1.0)	0.04
Calcium (per 1 mg/dl)	1.57 (1.02–2.43)	0.04	1.53 (1.0–2.60)	0.1
Albumin (per 1 g/dl)	.41 (0.55–3.60)	0.5		
Dialysis vintage (per month)	1.003 (1.0–1.0)	0.3		
Calcium channel blocker	0.41 (0.18–0.93)	0.03	0.32 (0.12–0.83)	0.02
Vitamin D use	0.41 (0.19–0.93)	0.03	0.38 (0.15–0.97)	0.04
AACS	1.11 (1.04–1.19)	0.001	1.09 (1.01–1.18)	0.03
Pre-CVE (vs. No)	2.01 (0.9–4.4)	0.08		

Abbreviations: DM, diabetes mellitus; BP, blood pressure; AACS, abdominal aortic calcification score; IDH, intradialytic hypotension; CVE, cardiovascular event

**Fig 1:**
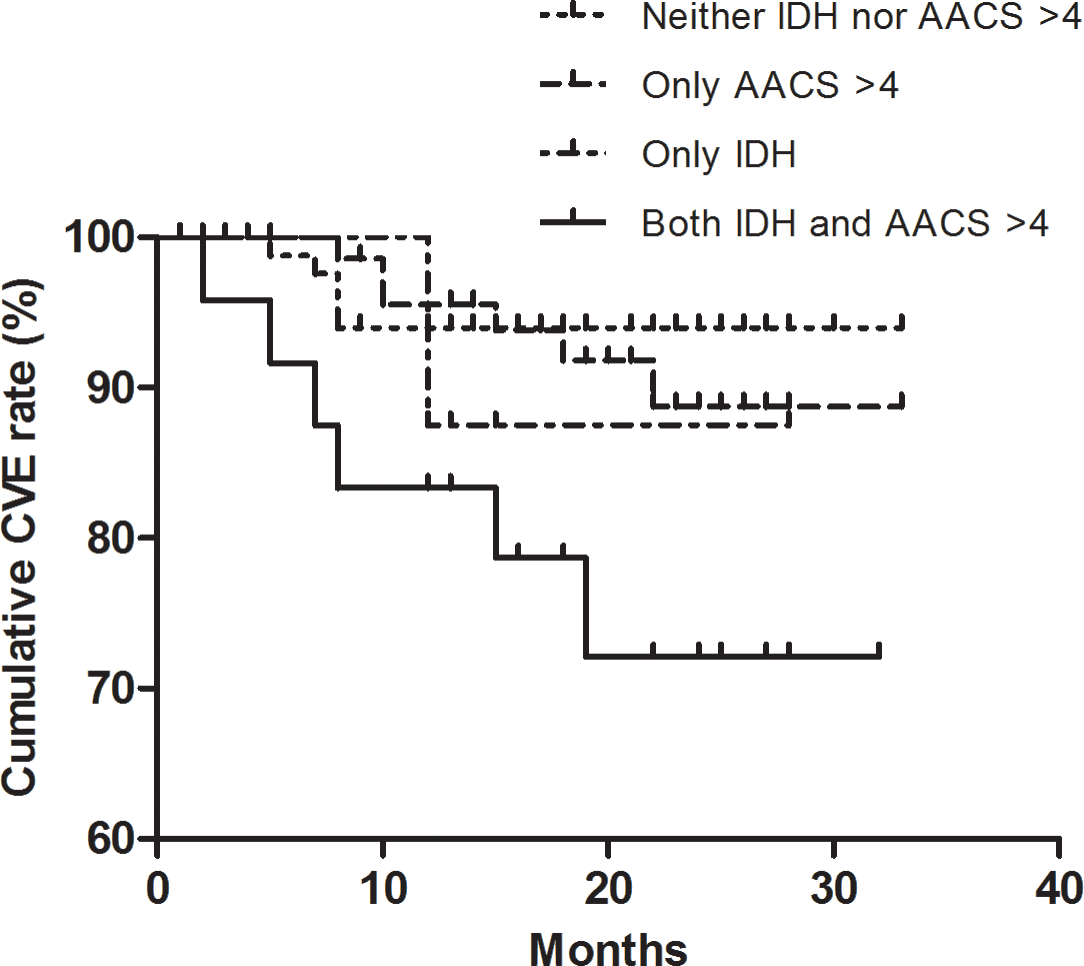
Cumulative rates of cardiovascular event. IDH, intradialytic hypotension; AACS, abdominal aortic calcification score.

The follow-up period was 18.3 ± 6.8 months. During follow-up, new CVE occurred in 18 subjects (9.4%) and 11 subjects (5.8%) died. Coronary artery disease occurred in 10 patients, peripheral vascular disease in 3 patients and cerebrovascular disease in 5 patients. The highest cumulative event rate for new CVE was observed in patients with both IDH and AACS ≧ 4 (27.9%, P = 0.008; Fig 1). Patients with either AACS≧ 4 or IDH had a 11.2%, and 12.5% cumulative CVE rate respectively, but the values were not significantly different from ones of patients with neither AACS ≧ 4 nor IDH (6%; P = 0.65 and P = 0.51, respectively). The cumulative event rate for mortality was not significantly different among the groups.

One hundred forty-three subjects and 159 subjects had PWV measurement and echocardiography, respectively. All subjects were divided into two groups based on the presence or absence of IDH. We also divided them according to AACS score. PWV value and echocardiographic parameters (LV ejection fraction, the peak mitral inflow velocities at the early (E), late (A) diastole, deceleration time (DT), early (E′) and late (A′) diastolic peak velocities, and E/E′ were compared between two groups. When comparing the presence or absence of IDH, only DT differed significantly (245.1 ± 78.2 vs. 210.5 ± 61.4, P = 0.01). Comparison of subjects with AACS ≧ 4 and AACS < 4 showed that PWV (2097 ± 633 vs. 1811 ± 331, P = 0.001), A (93.3 ± 25.8 vs. 85.5 ± 19.6, P = 0.03), E′ (4.4 ± 1.3 vs. 5.1 ± 2.0, P = 0.006), and E/E′ (20.4 ± 8.9 vs. 16.9 ± 7.3, P = 0.005) values were significantly different (Table 3).

**Table 3:** Pulse wave velocity and echocardiographic index according to vascular calcification.

Variables	AACS < 4	AACS ≧ 4	P- value
	(n = 94)	(n = 97)	
Pulse wave velocity	1811.4 ± 330.6	2097.4 ± 633.0	0.001
Echocardiac parameter			
LVEF (%)	56.5 ± 11.1	58.6 ± 9.0	0.183
LVMI (g/m^2^)	135.9 ± 41.6	142.4 ± 42.0	0.312
E (cm/sec)	77.1 ± 25.5	83.0 ± 26.2	0.263
E’ (cm/sec)	5.1 ± 2.0	4.4 ± 1.3	0.006
A (cm/sec)	85.5 ± 19.6	93.3 ± 25.8	0.031
E/A ratio	0.9 ± 0.4	1.0 ± 0.6	0.406
E/E' ratio	16.9 ± 7.3	20.4 ± 8.9	0.005
DT (msec)	214.6 ± 67.5	218.7 ± 64.3	0.694

Note: values are expressed as median ± SD

Abbreviations: LVEF, left ventricular ejection fraction; LVMI, left ventricular mass index; DT, deceleration time; AACS, abdominal aortic calcification score

Cox proportional analysis was used to evaluate risk factors for new CVE (see Table 4). In univariate analysis, age (hazard ratio (HR) = 1.06, 95% CI = 1.02–1.11, P = 0.005), previous CVE history (HR = 4.13, 95% CI = 1.36–12.56, P = 0.01), and IDH (HR = 3.30, 95% CI = 1.28–8.51, P = 0.01) were significantly associated with new CVE. The presence of both IDH and AACS ≧ 4 was a significant predictor for new CVE in univariate (HR = 3.53, 95% CI = 1.32–9.41, P = 0.01) and multivariate analyses (HR = 2.84, 95% CI = 1.04–7.74, P = 0.04). There were no significant predictive factors for mortality.0020

**Table 4:** Cox proportional hazard models for risk factors of new CVE.

Variables	Univariate		Multivariate	
	HR (95% C.I.)	p-value	HR (95% C.I.)	p-value
Age (per year)	1.06 (1.02–1.11)	0.005	1.05 (1.0–1.10)	0.04
DM (vs. No)	1.65 (0.62–4.40)	0.3		
Pre CVE (vs. No)	4.13 (1.36–12.56)	0.01	2.42 (0.75–7.88)	0.1
IDH+AACS>4 (vs. No)	3.53 (1.32–9.41)	0.01	2.84 (1.04–7.74)	0.04
Dialysis vintage (per year)	1.0 (1.0–1.0)	0.5		

Abbreviations: DM, diabetes mellitus; AACS, abdominal aortic calcification score; IDH, intradialytic hypotension; CVE, cardiovascular event

## Discussion

We have demonstrated that IDH is associated with vascular calcification. Patients with IDH had a higher AACS than did patients without IDH. AACS was a significant risk factor for IDH. Cumulative new CVE rate was higher in patients with IDH than without IDH, and highest in patients with both IDH and AACS ≧ 4. In multivariate analysis, having both IDH and AACS ≧ 4 was a significant predictor of new CVE.

Calcification of the coronary arteries and the aorta has recently been recognized as an important risk factor for cardiovascular disease in HD patients [10, 11]. Reduction of large-artery stiffness increases cardiac afterload [4]. Diastolic LV dysfunction is induced by increased afterload in a experimental study [12]. In HD patients, reduced large-artery stiffness may contribute to vascular calcification in the thoracic and abdominal aorta, independent of blood pressure (BP). Therefore, LV diastolic dysfunction may be associated with aortic calcification in HD patients. A study reported that diastolic LV dysfunction may be induced by increased vascular calcification and reduced arterial stiffness in chronic HD patients [5].

IDH is the result of not only cardiovascular system structural abnormalities but also of impaired vascular reactivity [13, 14]. Inadequate cardiovascular response to maintain adequate blood pressure in response to volume stress is a key issue in the pathogenesis of IDH in such patients [2]. HD patients with vascular calcification have a higher degree of arterial stiffness, which is associated with insufficient response to volume stress [4, 15]. Our results suggest an association between IDH with vascular calcification. However, we cannot explain the relationship between systolic and diastolic dysfunction, and IDH. This may be because the pathophysiology of IDH is multifactorial [16]. Meanwhile, vascular calcification was associated with diastolic LV dysfunction and arterial stiffness in this study, which is consistent with previous studies.

HD patients are at greater risk of cardiovascular (CV) disease-related morbidity and mortality compared with the general population [17]. The higher burden of CV disease-related events may be due, in part, to episodic tissue hypoxia resulting from IDH [18]. In this study, we found that presence of IDH was associated with new CVE. Presence of vascular calcification alone was not associated with CVE. However, coexistence of vascular calcification and IDH was an independent predictor of new CVE. Furthermore, the impact was increased in patients with both IDH and vascular calcification. IDH causes frequent ischemic episodes in the heart and arteries [18]. IDH also causes disturbance in achieving an adequate dialysis dose and substantial increases in calcium and phosphate product [19, 20]. Therefore, inadequate dialysis induced by IDH worsens VC in HD patients. In addition, patients with frequent IDH and with atherosclerotic arteries become vulnerable to ischemic injury.

Risk factors for IDH have not been defined clearly from the literature, but several conditions have been held responsible. Patients of advanced age (≥ 65), with diabetic nephropathy, CV disease, or autonomic dysfunction are at risk of developing IDH [16, 21]. Furthermore, clinical features such as low blood pressure levels before an HD session (< 100 mm Hg), poor nutritional status (hypoalbuminemia), and high ultrafiltration rate also predispose chronic dialysis patients to IDH occurrence [16, 21]. Our study results are comparable to the published data and, in this study; vascular calcification is shown to be an important risk factor for IDH in the adjusted multivariate model. Vascular calcification is related to functional disorders of the cardiovascular system including left ventricular dysfunction and peripheral artery malfunction, which may increase the risk of IDH [5, 22].

Our study has some limitations. First, there is the inherent weakness of all studies with a retrospective design; namely, the use of data from past medical records. Second, a number of patients did not undergo PWV measurement and echocardiography. Third, the follow-up period was short; studies of long-term prognostic implications of IDH and vascular calcification on CVE in HD patients are needed. Fourth, quantitative measurement of vascular calcification by computed tomography was not assessed. However, Kaupplia method was able to detect VC and predict CVEs [23]. In addition, our study did not consider the effects of oral medications to treat hypertension.

In conclusion, these results suggest that IDH is associated with abdominal aortic calcification and is an independent risk factor for IDH. Both IDH and high AACS were significant predictors for CVE occurrence. To improve mortality and morbidity in HD patients, a treatment strategy for reducing the frequency of IDH and calcification progression should be considered.

## Supporting information

S1 File The minimal data set. (XLSX)
